# The Differential Diagnosis of Two Cases of Chronic Periaortitis

**DOI:** 10.1155/2013/282067

**Published:** 2013-08-28

**Authors:** Takao Kato, Eri Minamino, Eisaku Nakane, Shoichi Miyamoto, Toshiaki Izumi, Tetsuya Haruna, Ryuji Nohara, Moriaki Inoko

**Affiliations:** Cardiovascular Center, Tazuke Kofukai Medical Research Institute, Kitano Hospital, 2-2-20 Ogimachi, Kita-ku, Osaka 530-8480, Japan

## Abstract

The imaging features of chronic periaortitis resemble those of infected aneurysms. Two illustrative cases of chronic periaortitis, in which the etiologies were caused by IgG4-related disease, are presented. The first case involved a 68-year-old man who presented with vague discomfort in his lower abdomen. The second case was a 42-year-old man who presented with a fever of 38°C and persistent, vague chest discomfort. Both cases demonstrated an increased amount of connective tissue around the aorta in computed tomography images and low intensity in the T2-weighed sequence and high intensity in the diffusion-weighed sequence, suggesting the presence of inflammation, in the magnetic resonance imaging. Negative blood cultures, elevated IgG4 levels, and pathological findings confirmed the diagnosis as chronic periaortitis due to IgG4-related disease. This is a newly recognized syndrome of unknown etiology, characterized by a fibroinflammatory condition, tumefactive lesions, and a dense lymphoplasmacytic infiltrate rich in IgG4-positive plasma cells. Both cases were successfully treated with corticosteroids. Infected aneurysms need to be carefully differentiated from this syndrome in view of the similar imaging features.

## 1. Introduction

IgG4-related disease is a newly recognized syndrome of unknown etiology that is characterized by a fibroinflammatory condition, tumefactive lesions, and a dense lymphoplasmacytic infiltrate rich in IgG4-positive plasma cells. Various symptoms are observed, according to the type of lesions involved [1]. Computed tomography (CT) imaging features of arterial lesions are characterized by homogeneous wall thickenings and enhancement in the late phases, after contrast infusion, corresponding to the presence of increased amounts of connective tissue, indicative of sclerosing inflammation [2]. Chronic periaortitis is one of the presentations of IgG4-related disease, and it resembles an infected aneurysm with a periaortic abscess [2–4]. The treatment choices for these 2 potential diagnoses are completely different, involving either corticosteroids or antibiotics [1, 5]. Here, we present 2 illustrative cases of chronic periaortitis, in which the etiology was an IgG4-related disease.

## 2. Case Presentations

**Figure 1:**
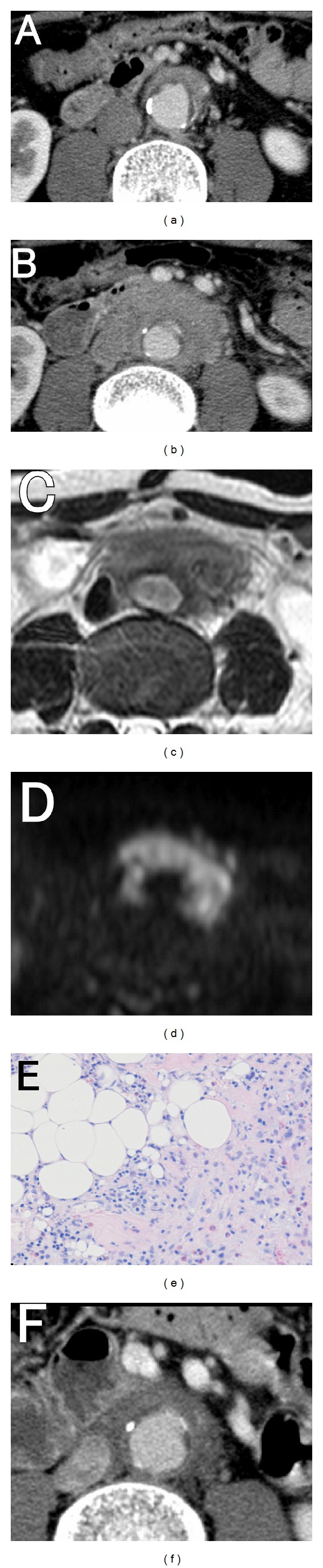
(a) A contrast-enhanced CT image, upon presentation. (b) A contrast-enhanced CT image two months after the presentation. (c) Low-intensity enhancement was observed in a T2-weighted, gadolinium-enhanced magnetic resonance image (MRI). (d) High-intensity was observed in the diffusion-weighted image. (e) A biopsy of the tissue revealed the infiltration of inflammatory cells and the absence of bacteria. (f) A contrast-enhanced CT image taken 3 weeks after starting the glucocorticoid therapy.

The first case involved a 68-year-old man who presented with vague discomfort of the lower abdomen. He did not have a significant past medical or family history and was not taking any medication.  Figure 1(a) shows a contrast-enhanced CT image taken upon presentation. Two months later, his symptoms remained unchanged, with a C-reactive protein concentration of 8.5 mg/dL and 7800 white blood cells/*μ*L, but without fever. Another CT was performed (Figure 1(b)) that showed increased connective tissue around the aorta. The area appeared to have low-intensity enhancement in a T2-weighted, gadolinium-enhanced magnetic resonance image (MRI: Figure 1(c)), and a high-intensity lesion in the diffusion-weighted image (Figure 1(d)). The patient's blood culture was negative, and his erythrocyte sedimentation rate was increased to 67 mm/h; his serum IgG4 levels were also elevated to 43.6 mg/dL. The differential diagnoses included chronic periaortitis with retroperitoneal fibrosis, malignant lymphoma, or another malignancy. A biopsy of the tissue revealed the infiltration of inflammatory cells and the absence of bacteria (Figure 1(e)). He was diagnosed as having chronic periaortitis with retroperitoneal fibrosis. He was started on 60 mg/day of prednisolone and gradually tapered off the drug.  Figure 1(f) was taken 3 weeks after starting the glucocorticoid therapy.

**Figure 2:**
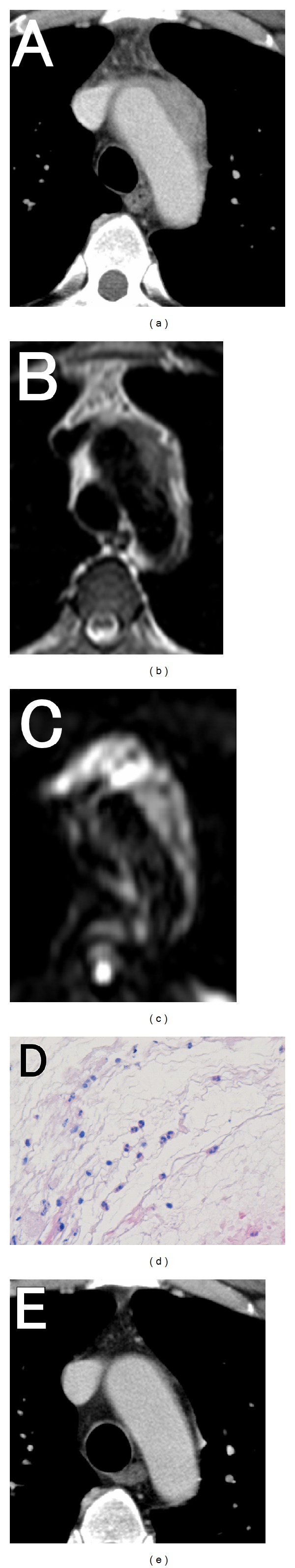
(a) Contrast-enhanced CT imaging showed diffuse thickening of the aortic arch, with enhanced mediastinal connective tissue. ((b), (c)) Gadolinium-enhanced MRI showed low intensity in the T2-weighted sequence (b) and high intensity in the diffusion-weighted sequence (c), suggesting the presence of inflammation. (d) A biopsy of the tissue, using a mediastinal scope, revealed infiltration by inflammatory cells, but no bacteria. (e) A contrast-enhanced CT image 2 weeks after starting the glucocorticoid therapy.

The second case was a 42-year-old man who presented with a fever of 38°C and persistent, vague chest discomfort. He, also, did not have a significant past medical or family history and was not taking medication. The patient's laboratory data showed a white blood cell count of 10,100/*μ*L, C-reactive protein of 9.64 mg/dL, IgG4 of 1964 mg/dL, and a negative blood culture. Contrast-enhanced CT imaging showed diffuse thickening of the aortic arch, with enhanced mediastinal connective tissue (Figure 2(a)). Gadolinium-enhanced MRI showed low intensity in the T2-weighted sequence (Figure 2(b)) and high intensity in the diffusion-weighted sequence (Figure 2(c)), suggesting the presence of inflammation. His differential diagnosis at that point included chronic periaortitis with mediastinitis; granulomatous inflammation such as tuberculosis or sarcoidosis; or malignancy. A biopsy of the tissue, using a mediastinal scope, revealed infiltration by inflammatory cells, but no bacteria (Figure 2(d)). He was diagnosed as having chronic periaortitis with mediastinitis. The patient was treated with 60 mg/day of prednisolone and was gradually tapered off the drug.  Figure 2(e) was taken 2 weeks after starting the glucocorticoid therapy.

## 3. Discussion

When these diagnoses were made, the most important, but difficult, task was differentiating between chronic periaortitis and infection. Because of the increasing population of elderly patients, the number of infected aneurysms is also on the rise, which is probably the result of endothelial damage of the great vessels [4, 5]. After obtaining imaging results, the next step in diagnosing chronic periaortitis is to confirm that the blood cultures are negative. This allows the healthcare provider to rule out the possibility of an infected aneurysm, for which antibiotic treatment would be the first choice [6, 7].

**Figure 3:**
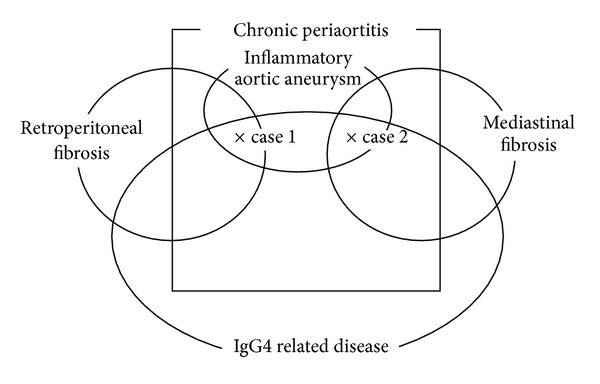
A schema for considering the possible overlaps in the current 2 patients.

Chronic periaortitis is characterized by a fibroinflammatory condition of the aorta [2, 3]. Some of the etiologies of chronic periaortitis are thought to be an inflammatory aortic aneurysm [3], IgG4-related disease [3], idiopathic retroperitoneal fibrosis [8], a manifestation of systemic autoimmune diseases [9], and a condition secondary to drug use or malignancy [8]. The disease entities cannot be clearly divided, and some overlap has been considered to exist. In this paper, we present a schema for considering the possible overlaps in the current 2 patients (Figure 3). With regard to IgG4-related disease, approximately 70% of patients demonstrate elevated serum IgG4 concentrations, which assist in making the diagnosis [1]. In addition, pathological examinations can confirm the IgG4-related disease by the presence of tumefactive lesions and dense lymphoplasmacytic infiltrates, rich in IgG4-positive plasma cells [1]. These observations are made in the absence of detectable bacteria, granulomatous lesions, or malignancies.

The imaging features of contrast-enhanced CT in patients with chronic periaortitis are characterized by homogeneous wall thickenings and late phase MRI enhancement, after contrast infusion [1, 2]. These observations accompany an increase in connective tissue that is indicative of sclerosing inflammation, sometimes with adhesions to the surrounding organs. These features also resemble those associated with infected aneurysms. 

However, infected aneurysms are usually based on atherosclerotic lesions; hence, calcification of the endothelium and a disruption of aortic wall calcification are often observed in conjunction with soft tissue inflammation and periaortic fluid or air collection [4, 10, 11]. MRIs are additional diagnostic tools. The T2-weighted images or mixed T1/T2-weighted short TI inversion recovery images are able to image the edematous lesion. The diffusion images can detect fluid collection, and gadolinium enhancement indicates an increase in inflammatory connective tissue [11]. Although edema and inflammatory connective tissue are common features of inflammation, these appearances assist in making the differentiation between the infected aneurysm and chronic periaortitis. Pathological findings or cultures from the pathological specimens confirm the diagnosis.

## 4. Conclusions

Although careful attention should be paid to differentiate them from infected aneurysms, chronic periaortitis can be properly diagnosed and effectively treated.
